# Oral Squamous Cell Carcinoma with an unusual clinical manifestation: a case report

**DOI:** 10.1186/1757-1626-2-6608

**Published:** 2009-04-20

**Authors:** Farnaz Falaki, Zahra Delavarian, Atessa Pakfetrat, Nooshin Mohtasham, Shiva Shirazian

**Affiliations:** 1 Department of Oral Medicine, Faculty of Dentistry and Dental research centerVakilabad Blvd, Mashhad, postal code:91735Iran; 2 Department of Oral Medicine, Faculty of Dentistry and Dental research center, Mashhad University of Medical Sciencespostal code:91735Iran; 3 Department of Oral and Maxillofacial Pathology, Faculty of Dentistry and Dental research center, Mashhad University of Medical SciencesMashhad, postal code:91735Iran; 4 Specialist in Oral MedicineNo.32-Shariati street-Tehran-Iran

## Abstract

**Introduction:**

Squamous cell carcinoma is the most common malignant tumor of the oral cavity and one of the 10th most common causes of death. It arises from dysplastic oral squamous epithelium. By considering the pathogenesis of squamous cell carcinoma, the smooth and intact surface for this lesion is not usual.

**Case presentation:**

A painful nodular lesion with smooth surface on the left buccal mucosa of a 75-year-old female patient was observed. She noticed it 2 weeks ago. Histopathological examination revealed oral squamous cell carcinoma.

**Conclusion:**

In this paper, we report an unusual clinical presentation of oral squamous cell carcinoma in buccal mucosa which is very rare.

## Introduction

Approximately 94% of all oral malignancies are Squamous cell carcinoma (SCC). The annual incidence and mortality rates vary between different races, genders, and age groups. In the United States this is 7.7 per 100,000 [1].

Like other carcinomas, the risk of intra oral carcinoma increases with increasing age especially for males [1-4]. Persons with oral SCC almost have been aware of an alteration in that site for 4-8 months before seeking professional help. There is minimal pain during the early growth phase and this may explain the delay in seeking professional care [1].

If the health care professional does not have a high index of suspicion, additional several weeks or months may elapse before a biopsy is performed [1].

Oral SCC has various clinical presentations such as exophytic, endophytic, leukoplakic and erythroplakic, which all of them show visible changes in the surface.

In the present paper, we report a case of exophytic oral SCC with a smooth surface which is an unusual presentation [1,5].

## Case presentation

A 75-year-old Caucasian female of Iranian nationality was admitted to the Department of Oral Medicine in Mashhad Dental Faculty with chief complaint of a painful mass in the left buccal mucosa, which was first noticed by the patient 2 weeks earlier and gradually increased in size.

**Figure 1.**
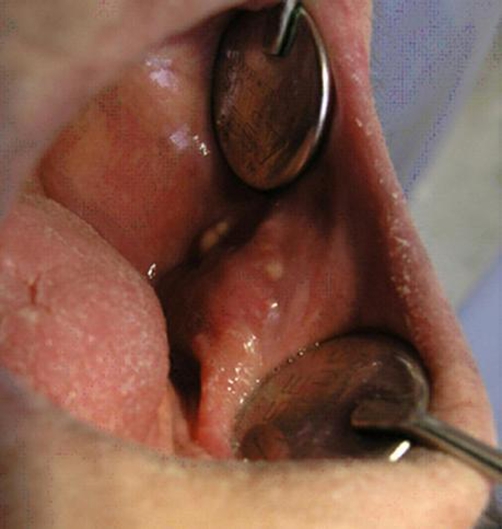
clinical view of the exophytic lesion with smooth surface in the buccal region. Small yellow papules are visible at the surface.

Intra-oral examination revealed a painful normal-colored nodular lesion with smooth surface in the left buccal mucosa with small yellow papules at the surface with approximate size of 2.5 × 1.5 cm and firm in consistency (Figure 1).

The patient did not feel any sensory changes in the affected area. Physical examination revealed no lymphadenopathy in submandibular or other neck triangles.

The patient was diabetic (type II). She did not have any risk factor for SCC (smoking or alcohol consumption) and have had 4 pregnancies.

By considering the characteristics of the lesion and our physical examination findings, our differential diagnosis were minor salivary gland tumors and other tumors of mesenchymal origin.

**Figure 2.**
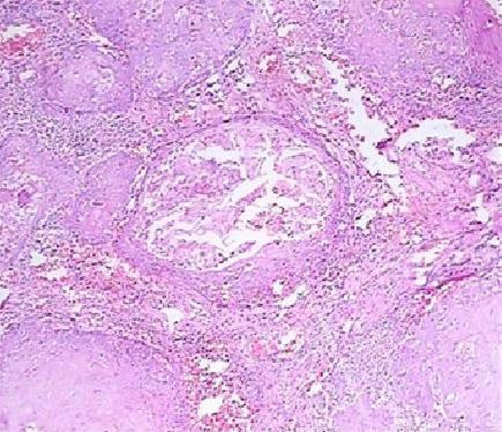
Invasion of tumor cells to connective tissue, Magnification 40 X, Hematoxyllin & Eosin staining.

Incisional biopsy under local anesthesia was performed and the specimen was submitted for histopathological examination, which revealed a malignant neoplastic proliferation of stratified squamous epithelial cells as sheets or islands of cells, invading to the connective tissue (Figure 2).

**Figure 3.**
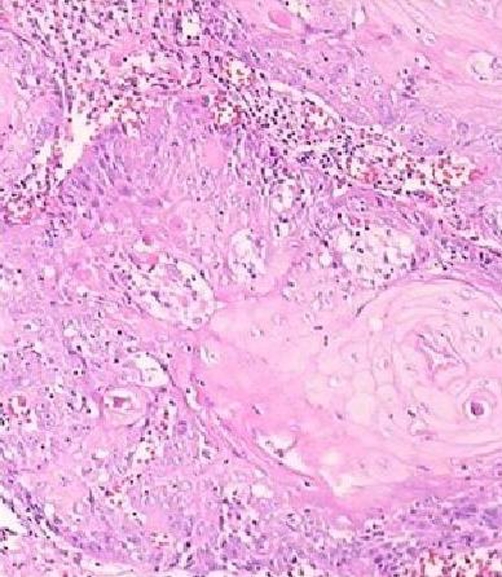
Pleomorphism, Hyperchromatism, and keratin formation in tumor, Magnification 100 X, Hematoxyllin & Eosin staining.

**Figure 4.**
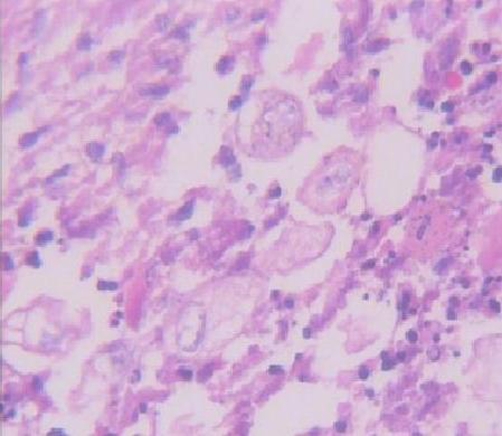
Pleomorphism and invasion of tumor cells, Magnification 400 X, Hematoxyllin & Eosin staining.

**Figure 5.**
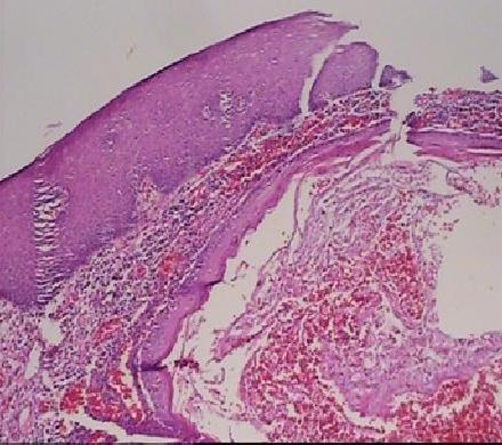
Smooth and intact epithelium of the lesion, tumor cells in connective tissue, Magnification 40 X, Hematoxyllin & Eosin staining.

The definite diagnosis was squamous cell carcinoma (Grade I). The patient was referred to an oncology department where surgery by using intra oral approach was performed and diagnosis of SCC was reconfirmed, but she died 3 days after surgery because of poor management of diabetes.

## Discussion

This is an unusual case of oral SCC in a 75-year-old female patient with a nodular lesion having a smooth and intact surface.

By considering the pathogenesis of SCC all presentations are associated with changes in the surface as expected for epithelial lesions and all exophytic SCCs have a rough surface and irregular shape.

On very rare occasion, squamous cell carcinoma may commence at a small location on the surface, burrow and undermine the subepithelial tissue in such a manner that the lesion appears mostly as a smooth surfaced exoplytic lesion, which makes a diagnostic challenge [6]. To the best of our knowledge it is the only reported case of oral SCC with smooth and intact surface reported in the literature.

The purpose of this article is to emphasize that even in smooth-surfaced rapid-growing oral lesions, SCC should be considered in differential diagnosis and this needs a careful examination and management by both medical and dental practitioners.

## Abbreviation

SCC: Squamous cell carcinoma

## References

[bib-001] Nevile BW, Damm DD, Allen CM, Bouqout JE (2002). Oral & Maxillofacial Pathology.

[bib-002] Greenberg MS, Glick M (2008). Burket's Oral Medicine.

[bib-003] Seoane J, Warnakulasuriya S, Varela-Centelles P, Esparza G, Dios PD (2006). Oral cancer: experiences and diagnostic abilities elicited by dentists in North-Western Spain. Oral Disease.

[bib-004] Lawoyin JO, Lawoyin Do, Fasola Ao, Kolude B (2005). Intra-oral squamous cell carcinoma in Nigerians under 40 years of age: a clinicopathological review of eight cases. Afr J Med Med Sci.

[bib-005] Regezi JA, Sciubba JJ, Gordan RCK (2008). Oral Pathology.

[bib-006] Wood NK, Goaz PW (1997). Differential Diagnosis of Oral and Maxillofacial Lesions.

